# Academia's Role to Drive Change in the Orthotics and Prosthetics profession

**DOI:** 10.33137/cpoj.v4i2.36673

**Published:** 2021-09-21

**Authors:** GF Kogler, CF Hovorka

**Affiliations:** 1 Orthotics and Prosthetics Unit, Kennesaw State University, Kennesaw, USA.; 2 Orthotics and Prosthetics Program, Department of Rehabilitative Sciences, East Tennessee State University, Johnson City, USA.

**Keywords:** Orthotics, Prosthetics, Education, Curriculum Reform, Healthcare Economics

## Abstract

This position paper outlines the important role of academia in shaping the orthotics and prosthetics (O&P) profession and preparing for its future. In the United States, most healthcare professions including O&P are under intense pressure to provide cost effective treatments and quantifiable health outcomes. Pivotal changes are needed in the way O&P services are provided to remain competitive. This will require the integration of new technologies and data driven processes that have the potential to streamline workflows, reduce errors and inform new methods of clinical care and device manufacturing. Academia can lead this change, starting with a restructuring in academic program curricula that will enable the next generation of professionals to cope with multiple demands such as the provision of services for an increasing number of patients by a relatively small workforce of certified practitioners delivering these services at a reduced cost, with the expectation of significant, meaningful, and measurable value. Key curricular changes will require replacing traditional labor-intensive and inefficient fabrication methods with the integration of newer technologies (i.e., digital shape capture, digital modeling/rectification and additive manufacturing). Improving manufacturing efficiencies will allow greater curricular emphasis on clinical training and education – an area that has traditionally been underemphasized. Providing more curricular emphasis on holistic patient care approaches that utilize systematic and evidence-based methods in patient assessment, treatment planning, dosage of O&P technology use, and measurement of patient outcomes is imminent. Strengthening O&P professionals' clinical decision-making skills and decreasing labor-intensive technical fabrication aspects of the curriculum will be critical in moving toward a digital and technology-centric practice model that will enable future practitioners to adapt and survive.

## INTRODUCTION

In this position paper, we describe the important role academia plays in shaping the orthotics and prosthetics (O&P) profession. In this approach, we present the challenges and proposed strategies for academia to prepare the next generation of professionals to continue to evolve and define the value of O&P care. This process will require future O&P professionals to embrace and integrate data driven approaches including new and emerging technologies as a therapeutic treatment for habilitation and rehabilitation.

## FACTORS INFLUENCING CHANGE IN HEALTHCARE

The profession of O&P, like many health professions, is under intense pressure to provide cost-effective treatments and quantifiable health outcomes. In the United States, where healthcare expenditures represent nearly 18% of gross domestic product ^1^ healthcare is confronted with an impending paradigm shift. For the profession of O&P this translates into several challenges such as the provision of services for an increasing number of patients by a relatively small workforce of certified practitioners delivering these services at a reduced cost with the expectation of significant, meaningful and measurable value (e.g., clinical outcomes). Pivotal changes are needed in the way O&P services are provided. To remain competitive, the O&P profession will need to move away from the traditional labor-intensive manufacturing processes and the typical clinical patient care processes based on anecdote, trial and error. Historically, these processes, which were once accepted when reimbursement for O&P services by third party payers were less rigorous, but they are no longer acceptable or sustainable in modern healthcare. Hence, there is a need for the O&P profession to adopt more efficient methods that utilize a systematic and quantifiable framework. Emerging evidence in manufacturing of O&P devices suggests that employing computer-augmented approaches, such as digital shape capture and additive, 3D-printing manufacturing methods, may soon make obsolete devices created by hand craftsmanship.^2^ In addition, the implementation of data science to inform evidence-based clinical decision-making suggests these methods may lead toward improved clinical outcomes and patient value.^3,4^ Other challenges to the O&P profession include a looming workforce shortage;^5^ increased patient volumes; more complex patients, whose care must include consideration of multiple diagnoses; underrepresented billing codes and insurance practices that require evidence of efficacy^6,7^ and proof of values-based care^8^ with no path for reimbursement. If the practitioner workforce deficit cannot meet the demand for services, alternative methods will likely emerge from related medical specialties or leveraged by new business models such as direct-to-consumer orthoses and prostheses. To stay ahead of these challenges, O&P education must build evolving curriculum models that can equip students to evolve along with a rapidly changing technology-driven healthcare environment. An emphasis on subject areas such as 3D modeling/printing, data science, and digital diagnostics (e.g., biomedical sensing) will provide students with a familiarity sufficient for them to “use it, interpret it and explain it”,^9^ and to understand their impact on decision making and treatment interventions.^9^

## PREPARING FOR CHANGES THROUGHOUT A PROFESSIONAL'S LIFESPAN

The academic programs that train future professionals in clinical practice, research, and education can fundamentally influence whether the O&P profession's scope of practice expands or contracts, and its ability to adapt to the factors that drive change. Emerging technologies, market forces, regulatory policy, and economic costs are transforming all sectors of healthcare, and these factors have already started to disrupt current practice. To cope with these demands, academic programs in O&P will need to reimagine their curriculum beyond the existing scope of practice in O&P, and will need to work with accreditation agencies (In the US, the Commission on Accreditation of Allied Health Education Programs and its committee on accreditation, the National Commission on Orthotic and Prosthetic Education, as well as relevant international organizations) to re-examine and update the core curriculum requirements of the master's degree^10,11^ along the lines we have suggested, expanding both graduates' skills and their ability to adapt to future shifts in the delivery of O&P patient care. Well-informed analyses of trends in medicine, healthcare, business, computer science, and manufacturing often provide useful forecasts for changes in these fields, and may also provide strategic perspective for O&P educators.

## BRIEF HISTORY OF ORTHOTICS AND PROSTHETICS EDUCATION

In the United States, O&P is evolving from its historical roots as an industry consisting of highly skilled “tradespersons” (i.e., technicians, fitters) to an expanded recognition as an allied healthcare profession. Clinical practitioners in O&P possess an entry-level master's degree and are supported by practitioner assistants and fabricators. Over the decades, the evolution from technician to clinician has required updates in the “tools of practice,” from device-centric, hand-crafted fabrication and fitting to contemporary practice involving greater emphasis on holistic, patient-centered care. This transition involved shifting the focus to clinical diagnostics, patient goal planning, treatment formulation, problem-solving and solution-based patient management. The shift from technician to clinician required a change in the curriculum which continues to evolve today. Academic preparation in the clinical sciences (e.g., body systems pathology and clinical conditions, methods of structured patient assessment, and clinical decision-making), materials science, and movement sciences were added to the necessary proficiency training in skills such as custom device manufacturing. Despite practice analysis data that defines 90% of today's practitioners as engaged in clinical rather than technical fabrication duties,^10^ educational programs continue to devote considerable time to device fabrication skills. Urgent curricular changes are needed to prepare the next generation of practitioners as the “tools of practice” in O&P move from hand fabrication to digital technologies that enable more efficient, economical, and adaptable processes that can better support contemporary healthcare delivery systems. More expedient and efficient device delivery will be needed to manage a greater number of patients and to cope with shrinking reimbursement for services. This increased demand will likely prompt practice managers to adopt the use of more prefabricated, custom-fitted, and modular O&P systems, and to consider the advantages that 3-D shape capture and additive manufacturing may offer.^2,12^ In exchange for the time saved by improved fabrication efficiencies, practitioners may be able to devote more time developing clinical patient management skills in areas that have traditionally been less well represented such as structured patient assessment and clinical diagnostics, goal setting, treatment planning and assessment of clinical outcomes.

Curriculum reform that includes technologies that are readily transferable to O&P and that complement clinical practice courses are strongly justified, according to Thimbleby^13^ who states that “technology drives healthcare more than any other force”. Ideally, one would hope that the graduates of O&P programs are empowered to become the next innovators that advance the profession and thereby dictate, in part, the direction in which the profession evolves. The domains of practitioner competency that exist today will have to evolve if we are to secure our role as healthcare providers of habilitation and rehabilitation using technologies as part of the plan of care.

If technology is not integrated with O&P, clinical acceptance may be hindered. For example, computer-aided-design and manufacture (CAD-CAM) has been used in O&P for over 40 years,^14^ especially in prosthetics, yet the use of CAD-CAM in clinical orthotics practice is still relatively small compared to conventional fabrication of positive model creation, model rectification, thermoforming, lamination, and other processes. While we acknowledge that a generation of clinic owners/managers/and decision-makers may have missed formal education in CAD CAM methods, still there has been little pressure in O&P curricula to strengthen students' computer modelling skills beyond an introduction to industry specific software.

## CHALLENGES IN SUSTAINING ORTHOTIC AND PROSTHETIC EDUCATION PROGRAMS

Currently there are 13 Master of Science education programs accredited by the Commission on Accreditation of Allied Health Education Programs (CAAHEP), based upon the recommendation of the committee on accreditation, the National Commission on Orthotic and Prosthetic Education (NCOPE).^15^ However, the track record of sustaining O&P clinical practitioner education programs at U.S. universities is rather poor. Of the 22 clinical practitioner education programs developed since the 1960s, nine have been shuttered, representing a 41% closure rate. The dismal closure rate is alarming, and such vulnerability is particularly disconcerting given that knowledge and skills training are the core foundations of the profession. While there are a multitude of reasons for education program closures, the leading factors appear to be the loss of federal funding, the ongoing decline of state funding, high program operational costs, including extensive (and expensive) lab space and equipment requirements, and a dearth of qualified educators and researchers.^16,17^ Moreover, O&P enrollments are among the smallest in a university, delivering little economic value derived from tuition and fees (e.g., Georgia Institute of Technology, St. Ambrose University, Rutgers University, Florida International University).^17^ How can the O&P profession advance and develop a cohesive, visionary, long term future with such a vulnerable funding model? The current O&P education curriculum needs to be re-examined if the profession is to address the challenges of limited budgets, substantial space and equipment requirements for labor-intensive fabrication and projected workforce demands.

Despite these barriers, four new education programs are planning to launch at universities across the U.S. in the next two years. These new programs have the opportunity to make the changes needed to ensure O&P programs remain viable in higher education, starting with curricular reform that reflects current and future O&P practice. In this case, greater emphasis should be devoted to areas in which today's O&P professionals have not had significant training, such as clinical diagnostics, identifying and prioritizing patient problems, treatment goal setting, formulation of treatment plan, dosage of O&P device use, prognosis, patient and caretaker communication and problem-solving skills. To counterbalance the increased emphasis on clinical knowledge and skill development, the technical component of the curriculum can be reduced by including new technologies that improve efficiency in fabrication processes, such as modular components that do not require custom fabrication. [We define technical fabrication as related to manufacture and production of devices, specifically thermoforming and lamination. Alignment, fitting adjustments and device assembly would be included in clinical skills.] Such changes are in line with contemporary practice analyses, devoting more time to preparing students for an imminent future of data-driven patient care, value-based care and a wide array of efficient fabrication technologies. With a significant reduction of the technical fabrication aspects of the curriculum, programs could replace those areas of the curriculum with streamlined manufacturing processes as well as digitally augmented clinical decision-making (e.g., data science and artificial intelligence) and problem-solving. This approach has the potential to strengthen and solidify the clinical value of O&P practitioners.

Evidence-based practice is probably the single most important — and widely neglected — element for driving improvements in both clinical decision-making and O&P curriculum. Expanding curriculum in this area, by including more coursework on searching, retrieving, evaluating, interpreting, and integrating new scientific knowledge will train students to utilize a systematic approach to patient care, using evidence to support their decision making and to quantify and characterize patient outcomes for values-based care. This approach reflects trends in evidence-based practice and values-based care that will likely continue into the future.

The concept of interdisciplinary and interprofessional collaboration is another critical curriculum addition. Due to the growing complexities of patient care and emerging technologies, it is no longer wise for clinicians to solve problems in a silo on their own;^18^ students must also learn these skills as part of their professional education. Just as interprofessional health care teams can leverage their collective intelligence for shared decision-making,^19^ engaging students from related programs (i.e., physical therapy, occupational therapy, engineering) in mock or actual patient case conferences can help shape their approach to patient-centered care^19,20^ and, help elevate the profile of O&P in the health care profession. Universities that support the interprofessional education concept are on the rise^21,22,^ and O&P programs will need to develop partnerships with multiple disciplines to capitalize on this emerging “value-added” curricular opportunity. An additional benefit of interprofessional education is that it encourages students to develop and practice good communication skills, a critical factor in understanding and communicating patient needs, priorities, and compliance, and for sharing knowledge and ideas among team members. Communication skills were ranked as the highest priority for O&P employers hiring residents in the United States.^23^ In addition, communication skills were identified as an area of needed development for the future of O&P care across the globe.^24^

How do we advance data-driven decision-making? You do this by strengthening a student's exposure to research in their clinical training. Graduates who understand and interpret the medical, allied health science, movement science and engineering scientific literature in a systematic and clinically relevant way will be trained to apply their knowledge correctly and directly, leading to more informed successful patient assessment and treatment. These practitioners of the future will instinctively weave the scientific method into their daily practice, formulating testable questions for patients, devising data collection protocols, then analyzing, interpreting, and applying the data they collect. In turn, this process will yield the evidence that defines value of care and justifies clinical treatment decisions.

## PROFESSIONAL AND TECHNOLOGICAL ENCROACHMENT

The increasing demand for O&P services, a projected shortage of providers, and market pressure for cost-effective treatments are creating opportunities for other health professionals to fill the needs unmet by O&P today. Moreover, technology-driven products and systems may yield an even greater challenge to the domain of O&P practice. Patient-consumers are prone to see an orthosis or a prosthesis as a “device” to address their needs, and may not fully appreciate the added value of an expert clinician A contemporary orthotist and prosthetist should manage the patient, identify their needs, and match them with the technology/device that will best meet those needs as part of a therapeutic “body motion control” treatment plan. However, the consumer-direct marketing of orthoses and prostheses at reduced cost will likely be perceived as an attractive option for future O&P users, particularly in circumstances where healthcare disparity and barriers to access are an issue.

For comparison, the profession of dentistry dealt with strikingly similar issues of professional encroachment over 40 years ago when the technical specialty of "denturists" successfully lobbied to provide dentures directly to patients, without a dentist and at significant cost savings.^25,26^ Alternative access to dental services continues to evolve with a 20-plus year history of consumer-direct marketing of mail-order options for dentures and orthodontia (i.e., teeth aligners).^27^ Consumers use home impression kits, photos, or scans for shape capture, which are then used to manufacture a person-specific fit of dentures or teeth aligners. The O&P profession is now experiencing the same trend, with consumer-direct prostheses and orthoses or print-your-own devices using a downloadable 3D file and 3D printer. Currently, there is limited data available on the impact or efficacy that the consumer-direct movement will have on the O&P profession, and on habilitation and rehabilitation in general but we consider it a real, albeit emerging, challenge.

Such encroachments into O&P are likely to advance and will be difficult to curtail. However, with diversely skilled practitioners and strategic business acumen, the O&P profession can influence what aspect of the market to uphold and preserve by building the next novel businesses in O&P. Academia can prepare future practitioners with the technical and business acumen to respond to the profession's service and market demands, addressing the entire spectrum of habilitation and rehabilitation in countries around the world.

## NEEDS AND PRIORITIES

The O&P profession can only estimate the future healthcare economic determinants and market impacts, but it can nonetheless strategically position itself for change. Because the demand of O&P services is expected to increase in tandem with a shortage of certified orthotists and prosthetists, more certified practitioners in O&P are needed. The principles of supply and demand cannot prevail without a stable environment for increasing the number of graduates entering the field; the expanded capabilities we suggest here, in clinical patient care and cost-effective manufacturing, can begin to curb the threat of other medical specialties competing for market share. But it is only with strategic curriculum changes that focus on strengthening skills in digital health technologies and evidence-based decision-making, that future O&P clinicians can confidently step into their role as "human interface experts” of wearables, exoskeletons and individualized assistive technologies. These reconceived practitioners will also be seen as valuable professional assets in interdisciplinary health care.

Clearly academicians and university programs cannot change the profession on their own. They will need strong allies for leadership and support, through partnerships with industry, corporations, businesses, and professional organizations to forge the path for the future of the O&P profession. Open, reflective partnerships with industry, corporations, and professional organizations will be critical to prepare the O&P profession for the future changes ahead. Foundations in evidence-based practice using digital diagnostics, data science and artificial intelligence that augment clinical decision-making are the primary technological changes expected across the entire healthcare sector. Therefore, our education programs must begin to strengthen the curriculum in these areas to create the new platform of skills and knowledge for practitioners. As a result, it is to be expected that clinical practice will change. Therefore, expanding a practitioner's skill set with three-dimensional computer modeling (3D CAD) could be a strategic advantage for the profession. Most allied health professionals do not possess 3D CAD computer skills and such a skill set would distinguish the O&P profession. The current clinical mastery O&P practitioners are renowned for, producing negative impressions and positive model rectifications to create custom-molded devices, could be replaced or augmented with new competencies in computer-based scanning, 3D modelling and manufacturing processes and techniques. The O&P profession has an opportunity to be proactive by preparing for the health economic changes that will alter clinical practice as we know it. The current students entering O&P programs are digitally and technologically savvy and as such, possess the skillset for this type of future and the new students expect it will be the same way they will practice in O&P. The profession must ensure that our future practitioners are empowered to respond to the forthcoming economic changes healthcare will impress upon medicine, habilitation and rehabilitation.

## CONCLUSIONS

Numerous healthcare economic factors are primed to provoke a major paradigm shift in the way O&P is practiced. Curricular reform and innovation are therefore needed for O&P education programs to ensure that the next generation of O&P professionals are empowered to integrate emergent and novel technologies within the span of their professional careers. Although machines can outsmart clinicians, they can't out-kind, out-humor or out-finesse them. Technology has a role in O&P, but only in the service of augmenting patient care. A critical aim is to ensure that an O&P professional's knowledge is distinct from other healthcare professionals and is regarded as clinically valuable. To lessen professional encroachment, O&P professionals will need to adapt to change and work toward becoming the primary innovators, to guide maturation of the field, and seek to expand the domain of practice. O&P education programs will continue to be at risk for closure due to economic burdens they impose. Strengthening clinical decision-making skills and decreasing the technical fabrication aspects of the curriculum will be important in moving toward a digital and technology-centric practice model while also making programs more sustainable and competitive in their respective universities.

## CALL TO ACTION

A “Visioning of the Future - O&P Summit” will be essential for academicians and community stakeholders to evaluate the trends in healthcare and the skills and knowledge needed for the next generation of practitioners. The last O&P summit to debate the future and draft a strategic plan for action took place 16 years ago.^16^ Orthotic and prosthetic communities of stakeholders should initially develop their own vision of the future, culminating in a comprehensive summit meeting whereby each community shares and debates their vision and followed by development of a strategic plan for the profession's future and its place in rehabilitation. Participating O&P communities would be represented as follows:

- Professional credentialing organizations (i.e., ABC) – The upcoming practice analysis of O&P professionals will inform trends in practice. The last practice analysis was conducted in 2015.
- O&P accreditation bodies (CAAHEP and NCOPE) would be careful listeners, to not bias the debate on new core curriculum updates for O&P clinical practitioner training and education.
- Industry – Practitioners and manufacturers will provide important perspectives to their needs and concerns.

A Visioning of the Future - O&P Summit would serve as the culminating meeting to set the strategic educational plan for the future. The process is critical because the profession needs ownership and unification to move forward.

## DECLARATION OF CONFLICTING INTERESTS

The authors declare there are no conflicts of interest.

## SOURCES OF SUPPORT

The authors declare no sources of external support for this project.
